# “I never expected that it would happen, coming to ask me such questions”:Ethical aspects of asking children about violence in resource poor settings

**DOI:** 10.1186/s13063-015-1004-7

**Published:** 2015-11-11

**Authors:** Karen M. Devries, Jennifer C. Child, Diana Elbourne, Dipak Naker, Lori Heise

**Affiliations:** London School of Hygiene and Tropical Medicine, 15-17 Tavistock Place, London, WC1H 9SH UK; Raising Voices, Plot 16, Tufnell Drive, Kampala, Uganda

**Keywords:** Child abuse, Violence against children, Ethics, Survey research, Low-income countries, Uganda, Corporal punishment, School violence, Sexual violence

## Abstract

**Background:**

International epidemiological research into violence against children is increasing in scope and frequency, but little has been written about practical management of the ethical aspects of conducting such research in low and middle-income countries. In this paper, we describe our study procedures and reflect on our experiences conducting a survey of more than 3,700 primary school children in Uganda as part of the Good Schools Study, a cluster randomised controlled trial of a school-based violence prevention intervention. Children were questioned extensively about their experiences of physical, sexual, and emotional violence from a range of different perpetrators. We describe our sensitisation and consent procedures, developed based on our previous research experience and requirements for our study setting. To respond to disclosures of abuse that occurred during our survey, we describe a referral algorithm developed in conjunction with local services. We then describe our experience of actually implementing these procedures in our 2012 survey, based on reflections of the research team. Drawing on 40 qualitative interviews, we describe children’s experiences of participating in the survey and of being referred to local child protection services.

**Results:**

Although we were able to implement much of our protocol in a straightforward manner, we also encountered major challenges in relation to the response of local services to children’s disclosures of violence. The research team had to intervene to ensure that children were provided with appropriate support and that our ethical obligations were met.

**Conclusions:**

In resource poor settings, finding local services that can provide appropriate support for children may be challenging, and researchers need to have concrete plans and back-up plans in place to ensure that obligations can be met. The merits of mandatory reporting of children’s disclosures to local services need to be considered on a case by case basis—in some places this has the potential to do harm. Research teams also must agree on what level of ancillary care will be provided, and budget accordingly. Further practical examples of how to address the challenges encountered in this work are needed, in order to build a consensus on best practices.

**Trial registration:**

NCT01678846 (clinicaltrials.gov), August 24, 2012

## Background

Violence against children is increasingly being recognised as an important area of public health and social policy research. Experiences of physical, sexual and emotional abuse and neglect during childhood are prevalent, and associated with numerous short and longer term health and developmental consequences [1, 2].

Surprisingly, there is a relative dearth of international, comparable epidemiological data on children’s experiences of violence, especially in low and middle income settings. However, researchers, policy makers, and donors are taking an active interest in this area. The United Nations Secretary-General's Study on Violence against Children specifically calls for more investigation to provide accurate and up-to-date prevalence, prevention and intervention data [3].

Gathering data on children’s experiences of violence is a complex task, and fraught with ethical and methodological challenges [4]. Most researchers consider asking young people to self-report their experience of violence using tools such as the ICAST-C [5] as the gold standard method for assessing abuse. Such tools, which use a series of questions about specific acts of violence, are considered to provide more accurate prevalence estimates than general questions on violence or reports by third parties. For example, one systematic review found that the average prevalence of retrospective self-reported abuse was 18 %, but that the average prevalence from parent/health care provider reports was only 4 % [6].

However, speaking to children themselves raises numerous issues [7], including the developmental capacity of children to understand the questions being asked [8]; their legal and developmental ability to provide informed consent or assent for participation in research [4, 8]; the unequal power relationships that exist between those collecting the data and child participants [9]; the duty of care of researchers towards children who disclose violence or other adverse experiences [10]; and the tensions between respecting a child’s confidentiality and any legal obligation that exists for researchers to report cases of suspected abuse [7, 11, 12]. There are additional considerations for the researchers employed to collect this data—hearing disclosures can result in vicarious trauma, which can be exacerbated if researchers believe children are not getting adequate services.

Even in high income settings, where well-structured and functioning child protection systems may exist, there is debate and a lack of clear guidance about conducting violence research with child participants. Special accommodations may need to be made where levels of literacy are low and child protection systems are not well developed. There are also risks, however, of not asking children about their experiences of violence [13]. Using third party reports or administrative data can yield inaccurate prevalence estimates and risk profiles, which could misinform prevention and response efforts. Not asking at all means that children are denied an opportunity to participate in a process that could lead to reductions in violence and improved outcomes for others like themselves.

There have been several reviews and reports on ethical issues involved in conducting research with children in resource-poor settings [10]. These documents outline some related theory and recommendations [14], but to our knowledge, there is nothing written about the actual process used to address ethical challenges in a large-scale survey or trial on violence against children in a low or middle income setting.

In this paper, we aim to provide a practical example of how current theory and best practice recommendations for conducting research with children may be applied to survey research, and to explore tensions between recommended best practices and real-life challenges encountered during data collection. Our specific objectives are to: 1) describe the procedures we developed for the baseline survey of a trial testing an intervention to prevent violence against children in Uganda; 2) describe our experiences of implementing these procedures during data collection, and 3) describe children’s perceptions of the research process using data from a qualitative study. We then 4) discuss our protocol and the children’s perceptions of the research in light of ethical principles.

## Methods

### Overview

The Good Schools Study (GSS) is a cluster randomised controlled trial (RCT) to evaluate the “The Good School Toolkit” developed by Raising Voices, a school-level intervention to improve children’s wellbeing and educational achievements by reducing violence against children in schools (NCT01678846, clinicaltrials.gov) [15, 16]. GSS has been approved by the Ethics Committee at the LSHTM and the Uganda National Council for Science and Technology Research Ethics Committee. The trial meets all standard requirements for initiation of trials in children, including that the trial could not be done in adults; the results of the research have the potential to produce real and direct benefit to children’s health; there is appropriate support in the case of unintended harm; and consent is obtained where feasible [17].

Our trial involves two cross-sectional surveys; we conducted the baseline survey in June to July 2012 and the endline in June to July 2014. Briefly, we randomly selected 42 primary schools in Luwero district and invited head teachers to participate; 100 % agreed. In each school, we invited all staff to be interviewed and a random sample of children in Primary Levels (P) 5, 6 and 7) (aged about 11–14 years). In total, we interviewed more than 3700 children and 500 school staff members at each survey. Implementation of the intervention started in October 2012 and finished in April 2014 in schools receiving the intervention.

### Consent procedures

We used a three-tiered consent process which is in agreement with the recent statement on ethical consideration for cluster RCTs [18, 19]. During the planning stages of the study, Raising Voices staff visited officials of the Ministry of Education and Sports at the national and district levels. A letter of support was received from the Ministry at the national level, and the District Education Officer in Luwero formally gave permission for the study to take place. Consent for participation was sought in person from head teachers for school participation in the cluster RCT and to approach parents and students for participation in research activities.

Parents had the opportunity to opt their children out of participating in survey research data collection either in-person, on the phone, or in writing at several time points. Information meetings were held at each participating school with a staff member from the school administration and a representative from the study team to explain the study to community leaders, and parents and guardians of children. In participating schools, each P5, P6, and P7 child also received a written notice to carry home to their parents or caregivers. These written notices and meetings emphasised that participation of children in research data collection was voluntary, that they had the opportunity to opt out of the study at any time, and they could choose not to answer any questions that they did not want to. Parents (and children) were told that some of the questions would relate to whether or not children had been hurt and that some children could potentially find this upsetting. They were also told that if the research team felt children were at risk of harm that they would be obligated to refer the child to local child protection authorities.

Individual students selected to participate in the survey were approached within their school, and informed consent was sought. The consent form was read aloud to each child, and contained a description of study procedures, and reminded children that they did not have to participate and had the right to stop the interview at any time. Children were also informed that if interviewers felt that their safety was at risk, that the interviewer would be obligated to discuss the case with a child protection officer responsible for child welfare locally (see Table 1).

**Table 1:** Wording in consent form about conditions under which confidentiality would be breached

*If you tell me about something that makes me think your current safety or wellbeing might be at risk, I may need to let the District Probation Officer or the health centre know so that I can do my best to keep you safe.*	

### Interviewer recruitment and training

We gave considerable thought to developing procedures to ensure interviewers were appropriately qualified to interact with children for our survey. We advertised for survey researchers with university degrees and asked for two references outlining previous work with children. In a developed country context we would have done a criminal record check, but this was not feasible in Uganda, so we relied on references instead. All interviewers attended 3 weeks of full time, intensive training, conducted in English and Luganda, the local language in the study area. The training included sessions on: a) violence against children and child rights; b) strategies to maintain privacy and confidentiality in a school setting where there may be onlookers; c) consent, including role plays to practice and practice relaying circumstances where we might have to consult child protection officials; d) techniques for building rapport and making children feel comfortable; e) role playing of interview techniques; f) practice sessions for handling disclosures of sexual violence; g) strategies for and the importance of remaining non-judgmental; and h) the study’s child protection protocol.

All interviewers were observed role playing and received individual feedback from senior research personnel. Interviewers were instructed to stop interviews if children became distressed or did not want to continue for any reason, and that their role was not to provide counseling. All interviewers signed a code of conduct detailing how they would interact with children, adapted from Save the Children’s code of conduct [20]. We clarified that any inappropriate actions with children would be grounds for dismissal. We trained an excess of interviewers and hired the best performers for fieldwork [21]. During the data collection process, the senior study team had daily debriefs with all supervisors and weekly debriefs with the larger team of interviewers. We discussed difficult cases and had a session for the larger team to discuss emotional reactions to the work.

### Data collection from child participants

All data on violence and mental health were gathered via face-to-face interview. Interviewers entered responses in real time into a pre-programmed mobile phone. Interviews took place in locations around the school grounds where interviewer-child pairs could be seen but not overheard. Male interviewers interviewed only male students; female interviewers interviewed male and female students. No incentives or reimbursements were provided for participation, to avoid inducement to participate, to avoid perceptions of unfairness at schools since not all children would necessarily be interviewed, and to avoid creating expectations that the intervention would provide compensation for participation. Interviews took place during the school day, both during breaks and scheduled class hours.

Experiences of violence were measured using the International Society for the Prevention of Child Abuse and Neglect Child Abuse Screening Tool-Child (ICAST-C) [5] and some items from the WHO Multi Country Study on Women's Health and Domestic Violence against Women (WHO Study) [22]. Students were asked about specific acts of violence (“Hit you with a stick? Caned you? Kicked you?) they had experienced from school staff, parents and others in the past week, past year, and ever in their life [16]. Interviews ended with scripted closures that differed depending on what the child disclosed (adapted from the WHO Study), with additional information for the child about whether or not we had to involve child protection officials and what would happen next if we did. All ended on a positive note to emphasise children’s strength and resilience (see Table 2 for an example).

**Table 2:** Scripted interview finish, for a child who disclosed severe violence

*I would like to thank you very much for helping us. I appreciate the time that you have taken. I realise that these questions may have been difficult for you to answer, but it’s is only by hearing from children themselves that we can really understand what it is like for children in school nowadays.*
*From what you have told me, I can see that you have had some difficult experiences. No one has the right to treat someone else in that way. However, from what you have told me I can also see that you are strong, and have been dealing with some difficult circumstances.*
*If you would like, I can make an appointment for you to see a counsellor. If you would like to talk further with someone about your experiences or situation, they would be happy to speak with you. It is completely free.*
*Because of what you’ve just told me I am concerned about your health and safety, so I'm going to have bring you to the health centre, to make sure that you are OK. I will also need to talk to somebody about this to make sure that you are safe, and so that we do whatever we can to make sure that this does not happen again. So what will happen next is that I will arrange for you to visit the local health centre, and I will also talk to the local child protection officer. It is this person’s job to make sure that children in your community are safe and taken care of, so they might want to speak to you as well. You can ask me what is going on and what is going to happen next at any time. How do you feel about that?*
*Are there any other adults you would like me to help you get in touch with about the things you have told me? Would you like me to inform your parents/caregivers or the head teacher? It is your choice.*
*If the child protection officer would like to get in touch with you about this, what is the best way for them to do that?*

In addition, we hired a counselor who had experience working locally with children. All children were offered an opportunity to speak with a counselor when they completed their interview, regardless of what they disclosed.

### Referring children who experienced violence

In addition to offering counseling to children via a study-employed counselor, we developed a comprehensive referral protocol to handle children who disclosed experiences of violence during the survey. This protocol was developed in consultation with local child protection experts and involved specific pathways of action depending on the severity and time frame of what the children disclosed (see Table 3). Referrals were deemed “urgent” requiring immediate intervention; “serious, but less urgent,” requiring action but accommodating slight delay; or “serious, but non-urgent.”

**Table 3:** Physical and sexual violence definitions used in GSS baseline survey to set response criteria for referring children^a^

Child discloses	Referral level	Indicated by positive answer to any of below discrete violent acts or injuries by any person	Response^b^	Time frame for appropriate response from lead response agency
Forced sexual intercourse within the past week, or obvious untreated physical injuries	1	In past week: threatened or pressured into sex; physically forced sexual intercourse or doing sexual things; suffered cuts, loss of consciousness; dislocated, sprained, fractured or broken bones; untreated injuries or severe injuries (requiring medical attention) reported as a result of physical or sexual violence; signs of acute malnutrition /neglect	Community Development Officer, leads referral, partner NGO child protection officer leads monitoring	Same or next day
	(defined as ‘urgent action needed’)			
Severe physical violence within the past week, or less severe sexual violence within the past week, or minor injuries observed	2	In past week: burnt; choked; cut with a sharp object; severely beaten; had genitals, breasts, or buttocks touched; exposed to pornographic imagery; forced undressing; exposed to nudity; forced to touch sb else’s genitals, breasts, or buttocks; involved in making of sexual photos or videos; forced kissing; suffered bruising; swelling; bleeding; difficulty sitting or walking; had to seek medical attention; and disclosures do not meet same urgency or severity criteria as for level 1 (e.g. forced sex or in need of urgent medical attention	As for referral 2	As soon as possible but up to four weeks since day child was interviewed and referred
	(defined as ‘less urgent but serious notification’)			
Severe physical violence within the past year or sexual violence within the past year, but no violence within the past week	3	As for referral 2, but past year; and disclosures do not meet same urgency or severity criteria as for level 2	Partner NGO child protection officer leads referral and reports to the probation office	Up to 12 weeks since day child was interviewed and referred
	(defined as ‘non-urgent but serious notification’)			
Severe physical violence, or any sexual violence before the past year	4	As for referral 2, but before past year; and disclosures do not meet same urgency or severity criteria as for level 2	As for referral 3	Up to 12 weeks since day child was interviewed and referred
	(defined as ‘non-urgent but serious notification’)			
No disclosure of specific violent acts in baseline survey but child says they wish to receive further help	0 (none)		As for referral 3	Up to 12 weeks since day child was interviewed and referred
	(defined as ‘voluntary notification’)			
All children offered counseling	N/A	Child requests counselling	Counsellor dispatched	No specified timeframe

^a^Refer to Fig. 1.^b^ Towards the end of data collection and following suspension of direct CDO notification, as part of alterations to the referral strategy, all referrals were directed to the partner NGO child protection officer and probation office for case management and monitoring

**Fig. 1:**
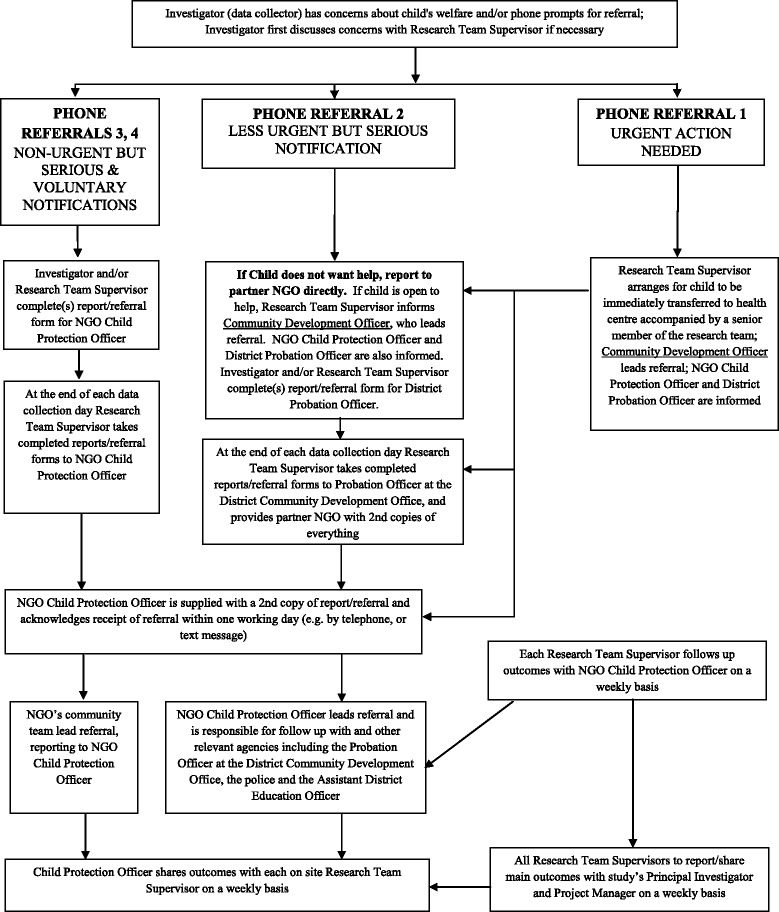
Flow diagram: Referral decision tree (from referral protocol)

For example, if the child disclosed that they had been raped in the past few days, they were referred immediately to the health centre for any necessary treatment, including post-exposure prophylaxis for HIV (see Fig. 1). If a child disclosed that they had been severely beaten in the past year, the referral was deemed “serious, but less urgent” and a local partner INGO was notified so that they could follow up according to their normal procedures. Referrals were prompted according to what children disclosed in response to different survey questions.

Decisions on what types of disclosures would necessitate referral, and where they should be referred were made considering legal requirements in Uganda and the structure of local child protection systems. In Uganda, community development officers are the first line of response to child protection cases at a local level. The most senior official responsible for child protection in the district is the District Probation Officer. In addition to these government entities, a variety of international and local NGOs provide child protection services.

The senior research team considered the possibility that a child might disclose experiences meeting our referral criteria, but be adamant that he or she did not want their experiences passed on to a third party. After extensive deliberations, we decided that such cases would be discussed anonymously with the District Probation Officer, who would decide the best course of action on a case by case basis. We took this decision in order to a) respect children’s wishes as far as possible and recognise that in most cases, they would be the best judge of their situation, but that there may be some circumstances where protection needs could supersede children’s stated wishes; b) because field researchers did not have the necessary training to make informed decisions on whether to refer children or not, and c) child protection decisions should be undertaken by a child protection professional in the local context. In practice, there was only one such case in the baseline survey, and it was decided that no further action needed to be taken.

## Results

For this section, we draw on quantitative data tracking referred children, and the observations of the Principal Investigator and Trial Manager, who oversaw the baseline survey in conjunction with senior Raising Voices staff in Uganda. During the baseline survey itself, interviewers reported that children understood the consent procedures, and understood that under some circumstances, their information might have to be passed on if they disclosed abuse. In most schools, interviewers reported no difficulty finding locations where interviews could take place within sight of others, but where others could not overhear; however in a minority of schools, this proved difficult. This tended to be in larger schools with more students, and those located in urban areas where there was less outside space available. Interviewers were instructed to wait until space became available, and data collection happened at a slower pace in these schools.

### Our experiences of referrals

Dealing with referrals—both constructing the strategy and actually implementing it—was by far the most challenging aspect of the baseline survey. Despite careful forethought and agreements with relevant actors (e.g. the District Probation Officer, community development officers and the local office of an International NGO partner (not Raising Voices)), the process of referral did not go smoothly. From the baseline survey, 529 children were referred as needing attention as per our protection protocol. Detailed information about follow-up and case management is provided elsewhere [23], but only 3.8 % of cases were followed-up by local community services within agreed time frames. In order to fulfill our ethical obligations to participants, the senior research team had to intervene, first to ensure that referrals were happening according to our strategy, and later to alter the strategy itself to prevent harm to children from inaction and poor responses from some community development officers. Given the poor response, the study team recruited our own study counselor (originally funded by Raising Voices to provide generic counseling post survey) to follow-up outstanding child protection cases.

## Children’s experiences

### Data for this section

We draw on our referral data collected for a qualitative study that we conducted with children after the baseline survey had taken place. This qualitative study involved purposive sampling of children who had been referred per our protection protocol and/or who had requested counseling as part of the baseline survey. We interviewed 23 children who had been referred and who had received some sort of help from a protection agent, another 7 children who had been referred and who also had requested counseling after the survey, and a further 10 who had been referred for violence but did not request counselling and did not receive any support from a community agency. The children attended different schools in Luwero. 22 were girls and 18 were boys, and most were aged 12–14 years.

These qualitative interviews focused on children’s experiences of the child protection system, but children also provided comment on their experience of the survey interview and the follow-up counselling. Interviews were conducted by a team of experienced violence researchers, in Luganda. A structured but open ended interview guide was used. All interviews were tape recorded and transcribed, and translated into English for analysis. The trial manager developed a coding framework and coded transcripts thematically, drawing on techniques from Grounded Theory including constant comparison and searching for deviant cases. NVivo was used to organise the data.

### Children’s experiences of interviews

Children’s accounts suggest that they understood the confidentiality promised them during the interview and that this was key to their decisions to disclose their experiences to the interviewers. Of the 529 children referred, about half had previously disclosed that they had experienced any violence (prior to the interview). On the whole, interviewers seem to be quite successful in developing and maintaining rapport with children, and children indicated that this was central to their decisions to disclose:

“I trusted him [the interviewer]…he behaved well…because he told me the reason he had come…that they are going to help children that have problems” (child 1); “I trusted him [interviewer]…because of the way he spoke to me…I was confident there would be no problem…the reason I trusted him…he introduced himself to me very well…and he assured me that no one else would know about it…apart from himself alone…so in my heart I got the idea to tell him…and I told him everything” (child 2).

Children were keen to point out that they didn’t disclose their problems to just anyone, once again emphasising the basis of trust that was necessary in order for disclosure to come about. Many children explicitly recall being reassured about confidentiality and it seems that this was a decisive factor in their decision to disclose. However, two children said they felt scared when talking to the interviewer because they feared that their answers would be passed on to the persons that they had exposed as perpetrating abuse against them.

Children's accounts also suggest that the main factor in their decision to disclose was the expectation of help, or in the hope that something would change and they would no longer be at risk of violence. In most cases, disclosure was in relation to cases of physical abuse from teachers; some were for sexual harassment from fellow students or strangers, and a few were direct requests for HIV testing:

(e.g. for cases of abuse from teachers: “I knew he/she [RV interviewer] would help me”; “I spoke knowing that this problem would be solved”; for cases of sexual harassment/rape from fellow students/strangers: “I wanted him/her [RV interviewer] to save me from this person”; “I wanted the situation to improve”).

The severity of abuse also played a role in the decision to disclose:

“I could not stand the beating any longer” (child 1); “I saw that my life was in so much danger” (child 2); “master was beating us a lot” (child 3); “I was mistreated” (child 1); “he/she [teacher] would beat me all the time” (child 2); “because of the pain I had gone through” (child 3).

Some children may have interpreted the phrasing in our consent form about ‘informing child protection officials if we think someone might hurt you’ as meaning that they would receive help:

“I realised that he/she [RV interviewer] would help me out of the problems that I had…according to the questions she asked me [reference to the baseline survey] and the words he/she told me at the beginning [reference to the consent form] (child 4).

On the whole, the overwhelming majority of children expressed relief about being able to discuss their experiences, and did not experience the interview as a traumatic event. Children valued being asked about their problems and expressed relief about being able to talk to someone about their experiences:

“I felt that the dirt that was on my heart had gone off”; “I felt that my heart was somehow relieved” (child 5)

Some even made direct reference to the survey questions about violence:

“…deep in my heart, I felt happy…because I never expected that it would happen…coming to ask me such questions” (child 6); “I felt good about the questions she was asking us” (child 7)

This was not universal however, and one child said that the interview had caused her to recall the pain of the original abuse. Several others mentioned that they felt ‘bad then good’ at the interview, and several children mentioned feeling scared about their information being passed on.

### Children’s experience of referrals

Of the 40 children in the qualitative study, 23 were sampled because their cases were referred to community agencies directly. However in 8 cases, community agencies responded but did not see children directly (instead speaking to headteachers for example). Only 15 children were actually seen by community agencies. The following section is based on their accounts. These 15 were generally happy with the response they received, even when that response fell below standards that the research team would deem acceptable.

Children reported mixed accounts of the initial approach of child protection staff. As part of the data collection at the time point of the survey, all referred children were asked how they would prefer to be re-contacted, and this information was passed along to child protection officers. Accounts suggested that some children were happy that someone was coming to see them, but others were at first worried and unclear as to what was happening:

“at first I was a bit worried…wondering where they were taking us and if they were not going to ask us more questions”. (child 8)

Although child protection officers asked for head teachers’ permission to call students from class, only a few children recalled being told which organisation the child protection officers came from. Although NGO staff explained their reason for coming and what was going to happen next, some children described being asked to get into cars and taken away without knowing where they were being taken (some children were taken for HIV testing):

“generally, those two [partner NGO CP officers] did not tell us anything…because they only got us from here…and put us in a car…we just left this place without them telling us anything” (child 9); “they asked permission from teachers to take us…we were three children…and we did not know where they were taking us” (child 10).

However, all of these children reported that they understood where they were being taken by the time they reached the medical centre.

Children reported that child protection officers generally took steps to protect confidentiality, by speaking quietly to prevent them from being overheard, or speaking to them away from others:

“they [other people] never heard, they were chased away…she [international NGO CP officer] was speaking at a low tone only for me to hear…we were under that mahogany tree” (child 8); “they told me not to tell anyone about it…that no one else would get to know about it” (child 9); “they told me…what we talked about should be kept confidential, that it should be between me and them” (child 2); “they [international NGO CP officers] refused us to tell the teachers” (child 3).

In most cases children perceived that these steps were effective. Children also reported that community agency staff came to visit them ‘not long’ after the initial interview for the baseline survey. Actual times varied widely, but one child who waited for more than one month reported that it ‘was not a long time to wait’.

Almost all children reported that they were happy with the response that they received. For most children, it seemed that someone simply coming to see them indicated that their concerns had been taken seriously and they felt that this was a positive result:

“there is nothing better than what she did because she took her time…and came....and saw me” (child 11)

Children’s positive perceptions were also linked with concrete outcomes of the visits by child protection officers:

“because I felt happy about what she [partner NGO volunteer] told me…that she would talk to them [child is referring to teachers who beat child] so that they could change what they were doing” (child1); “because they told me my status…they tested me and after testing me…they told me the truth…I got to know my true status”.

Besides changes to their situations that they perceived had resulted from visits by child protection officers, children were also largely satisfied with the manner in which child protection officers interacted with them:

“they never treated us badly” (child 1); “because they separated us and did not reveal the secrets of each child we went with” (child 2).

However, this was not universal. In general, children’s negative experiences as described by them were related to examples of extremely poor intervention and care by child protection officers. For example, a community development officer visited a child at home and, with the child’s permission, spoke to an abusive family member. The child reported that the abuse worsened, and that the contact number the officer had given her was not working. This child understandably reported a negative perception and experience of help from community agencies, and does not want help in the future. This child has been offered ongoing contact and support from our study counselor, but in the absence of this, it is unclear if her case would be further considered by local child protection services. This case illustrates some of the difficulties in considering where the research team’s duty of care and duty of rescue end.

## Discussion

We provide a practical example of designing and implementing ethical aspects of survey research on violence against children in a low income setting. Although we were able to implement much of our protocol in a straightforward manner, we also encountered major challenges in relation to the response of the local system to children’s disclosures of violence. Below we discuss three key issues in relation to the evolving literature on ethics of research involving children.

### Asking children about violence

Traditionally there has been reluctance to ask children directly about potential experiences of abuse for fear of causing psychological trauma or distress. However, few studies have found any evidence of negative mental health consequences [24]. This is consistent with studies among adult women, which likewise have found that women generally report feeling better after participating in a study on violence, especially those who disclosed abuse [22]. Similarly, our child participants largely reported disclosing their experiences as a positive experience. Mudaly et al. note that abused children in their small qualitative clinical study sometimes became upset during the research process, but after a full debriefing reported feeling positive [11]. Drawing on the WHO Multi-country Study on Domestic Violence and Women’s Health, we scripted our interviews to end on a positive note by focusing on the child’s strengths.

Similar to Jewkes et al., we did not conceal the subject of the research from the wider community [25]. In our case, school staff participated in a parallel survey and were asked questions about their experiences of and use of violence. Staff were not directly made aware that children were also being asked about their experiences, but it is reasonable to assume that content of the children’s survey became known. We must acknowledge the possibility that some children could have experienced some retaliatory violence, where school staff became aware that children had disclosed their experiences to the interview team. However, we judged that the risk of this was low. Since concealment would have been near impossible in practice, we judged that it was better to be open about the content to avoid giving the impression that any particular individuals had been singled out for participation as a result of being very violent or experiencing high levels of violence. It was also clear that all staff were asked to participate, and that large numbers of students were being randomly sampled. Although there is no way to accurately measure levels of retaliatory violence without doing another survey, as part of our monitoring strategy to examine the implementation of the Toolkit intervention, we had a research officer collecting monitoring data on a continuous basis from schools. Part of the monitoring data collection involved asking a selection of students and staff about their perceptions of increases or decreases in violence, and any recent incidents. We did not receive any reports of increased violence during the implementation period, and our overall trial result indicates a large reduction in physical violence from school staff towards students [26].

### Reporting of abuse to local child protection structures

In high-income countries, certain professionals are required by law to report known or suspected cases of abuse to child protection authorities. There is considerable debate, however, about the relative merits of reporting (or referral) in cases where the child vehemently rejects disclosure and reporting is not required by law. There is no consensus in the literature about the obligation to report [12], especially if the adequacy of the response cannot be assured. Some argue that when it is not clear that reporting will improve outcomes, respecting the child’s evolving autonomy and preserving confidentiality should take precedence [27].

Others, especially those influenced by a legal environment that requires disclosure, argue that reporting is ethically mandated by the principle of beneficence [27]. For example, Fisher argues that the moral obligation to protect children supersedes any cost-benefit analysis. “Simply put, reporting suspected abuse and neglect is the just thing to do.” [28] But as conceptualised here, beneficence would assume that the reporting leads to positive outcomes for the child, rather than stigmatisation, retaliation, or loss of willingness to trust adults in other future situations.

Our team itself was split over how best to discharge our ethical obligations to child participants in the study. Some members felt that the duty of care should always take precedence over concern for confidentiality; whereas others felt that as child rights advocates, we should allow children to be owners of their own information. Establishing which strategy is in the “best interests of the child” is further complicated by the weakness of local protection systems. In this study, reporting cases to local child protective services did sometimes result in consequences that we viewed as harmful, including stigmatising children by naming them publicly as “abused” and in one case, exposing them to retaliatory treatment from perpetrators who came to know of the child’s disclosure.

Significantly, however, children reported better, more supportive experiences with the counselor hired by the study; but we had not anticipated needing to use this individual to implement the response protocol.

### Duty of care

A key question in all research is where does the obligation of the investigators end, especially when working in settings with weak health and social service infrastructure? Traditionally, ethical guidance on clinical research maintained that researchers were only obligated to provide services or health care necessary to answer the question under study or to avoid or mitigate harm directly resulting from participating in the research [29]. More recently, ethicists have begun to rethink this construction, arguing instead that the duty of care and the duty of rescue can create obligations upon investigators to provide services beyond those immediately required for the study when they possess “expertise sufficient to meet the need safely and effectively, ability to apply that expertise without incurring inordinate costs, absence of other individuals or organizations able to meet the need, and freedom from competing obligations that preclude taking the action otherwise called for” [30].

We originally took the view that our obligation was to refer children to existing child protection services, but when this proved inadequate, we took it upon ourselves to ensure appropriate psycho-social support to the children by re-deploying the study counselor for this purpose. Attending to children’s health and emotional needs arising from the original abuse (as opposed to from participating in the study) is a classic example of providing “ancillary care” outside the immediate needs of the study. Given that there was indeed an “absence of individuals or institutions able to meet this need,” we concluded that the duty of care obligated us to attend to the immediate therapeutic and health needs of the children.

Given our experience, we considered whether we might have been better off to anticipate from the beginning that the study would provide follow up services directly, rather than rely on local child protection services. This would have eliminated the challenge of securing funding mid-stream to support several private counselors to fulfill these roles. At the same time, planning from the beginning to circumvent local child protection services fails to respect local sovereignty or strengthen local systems. International researchers should seek to work with local agencies where possible and shift to “Plan B” only when the well-being of children is at stake.

In Uganda, it is a legal requirement that research is approved by a national ethics committee (in addition to the committee at an international university). It may be helpful for local ethics boards approving research involving children to explicitly require researchers to consider the balance between duty of care obligations and a potential lack of local availability or capacity, and to provide guidance to researchers to do so. Nonetheless, it remains important that future researchers and donors remain aware that mid-stream course corrections may be necessary and that the project may need to sponsor additional support services. We would also suggest that it is essential that researchers budget appropriately for a “Plan B”, in case of difficulties.

### Limiting false expectations

There was some evidence from our qualitative work that children perceived that the research team would provide direct and immediate assistance to them as individuals. Our consent process did explain that the study was to help all children in schools, and did not make any specific statements about individual help, however it seems that among at least some children there may have been misconceptions. This likely relates to the ‘therapeutic misconception’ in clinical research, where participants believe that treatments they are receiving are designed with their therapeutic best interests in mind (that is, they are receiving individual help), rather than in line with a research protocol that benefits the broader good [31]. Wording on consent forms should include an explicit statement to make clear that there may not be any immediate individual benefit from participation. Our colleagues have used wording similar to “You will not benefit directly from participating in this study, but your participation will help others like you”, which may be appropriate in different settings.

## Conclusion

The ethical aspects of conducting epidemiological research on violence against children in resource poor settings are complex, and researchers must discuss and reflect on principles and best practices. Those conducting research on violence against children, with children, will almost always encounter cases of abuse, and clear protocols must be outlined before data collection begins on how these cases will be handled. Protocols should include precise definitions and referral pathways, and be developed considering the local legal and practice environments. Where services are not well developed, alternative strategies to support children should be agreed upon and detailed, and, as with all research, approved by local ethics boards. Based on our experience, mandatory reporting of cases to local services should be considered on a case by case basis, and the costs associated with ancillary care must be considered by donors and implementing agencies. However, given the scale of violence against children globally, these challenges should not dissuade investigators from conducting research and testing prevention and response strategies. Understanding both levels of violence and how children experience violence is essential to effective prevention and response. Further practical examples of how to address the challenges related to this work in diverse contexts are needed, in order to build a consensus on best practices.

## Abbreviations

ICAST-C: International Society for the Prevention of Child Abuse and Neglect Child Abuse Screening Tool-Child
RCT: Randomised Controlled Trial
GSS: Good Schools Study
WHO: World Health Organisation
HIV: Human Immunodeficiency Virus
NGO: Non-Governmental Organisation
CP: Child Protection
